# Glucocorticoid-Induced Leucine Zipper Inhibits Interferon-Gamma Production in B Cells and Suppresses Colitis in Mice

**DOI:** 10.3389/fimmu.2018.01720

**Published:** 2018-07-23

**Authors:** Stefano Bruscoli, Daniele Sorcini, Sara Flamini, Andrea Gagliardi, Francesco Adamo, Simona Ronchetti, Graziella Migliorati, Oxana Bereshchenko, Carlo Riccardi

**Affiliations:** ^1^Section of Pharmacology, Department of Medicine, University of Perugia, Perugia, Italy; ^2^Section of Hematology, Department of Medicine, University of Perugia, Perugia, Italy; ^3^Department of Surgery and Biomedical Sciences, University of Perugia, Perugia, Italy

**Keywords:** corticosteroid, glucocorticoid-induced leucine zipper, inflammation, IFN-γ, mouse models

## Abstract

Glucocorticoid-induced leucine zipper (GILZ) is transcriptionally upregulated by glucocorticoids (GCs) and mediates many of the anti-inflammatory effects of GCs. Since B cell activity has been linked to cytokine production and modulation of inflammatory responses, we herein investigated the role of GILZ in B cells during colitis development. B cell-specific *gilz* knock-out (gilz B cKO) mice exhibited increased production of the pro-inflammatory cytokine IFN-γ in B cells, and consequently CD4^+^ T cell activation. Increased IFN-γ production in B cells was associated with enhanced transcriptional activity of the transcription factor activator protein-1 (AP-1) on the IFN-γ promoter. Moreover, GILZ deficiency in B cells was linked to enhanced susceptibility to experimental colitis in mice, and this was reversed by administering GILZ protein. Interestingly, we observed increased production of IFN-γ in both B and T cells infiltrating the lamina propria (LP) of gilz B cKO mice. Together, these findings indicate that GILZ controls IFN-γ production in B cells, which also affects T cell activity, and increased production of IFN-γ by B and T cells in LP is associated with predisposition to inflammatory colitis in mice.

## Introduction

Glucocorticoids (GCs) have anti-inflammatory and immunosuppressive activities that involve nearly all branches of the inflammatory responses. Although GCs are potent anti-inflammatory drugs, their clinical effects are transitory and chronic use is accompanied by serious side effects that necessitate discontinuation of therapy (1, 2). Therefore, knowledge of novel mechanisms and the development of new drugs that can substitute for GCs may prove critical for suppressing inflammation.

The gene encoding glucocorticoid-induced leucine zipper (GILZ) is rapidly induced by dexamethasone (DEX), a synthetic GC (3, 4), and GILZ has been shown to mediate the anti-inflammatory activity of GCs (5–9). GILZ modulates the same immune response- and inflammation-related signaling pathways implicated in GC-induced anti-inflammatory and immunosuppressive activities, suggesting that GILZ-based strategies can constitute a new approach for the treatment of inflammatory/autoimmune diseases.

The murine *gilz* gene encodes a 137 amino acid (aa) leucine zipper (LZ) protein, which is almost identical to its human GILZ protein homolog (135 aa, 97% identity) (3). GILZ is composed of three domains comprising a transforming growth factor (TGF)-β-stimulated clone (TSC) box, a central LZ domain, and a proline (P)/glutamic acid (E)-rich (PER) region in the C-terminal part (10). Unlike most of LZ-containing proteins, GILZ does not contain a DNA-binding basic region. GILZ is mostly located in the cytoplasm, where it interacts with several signaling molecules and transcription factors including activator protein-1 (AP-1), a transcription factor pivotal for the activation of immune cells during inflammation (11). Indeed, GILZ heterodimerizes with both the c-Fos and c-Jun components of AP-1 (12), and over-expression of GILZ inhibits interleukin (IL)-2 production, a cytokine that plays a central role in T cell homeostasis and activation (4, 10, 13). Conversely, T cell activation suppresses GILZ expression (4, 13, 14), and this reciprocal inhibitory activity between T cell activation and GILZ expression indicates that GILZ modulates T cell activity, suggesting that altering GILZ expression may affect inflammatory processes such as inflammatory bowel diseases (IBDs). Indeed, we observed that over-expression of GILZ in T cells in GILZ transgenic (TG) mice induces downregulation of T helper (Th)-1 cells and upregulation of Th-2 cells (15, 16). This correlates with inhibition of pathogenic activity in CD4^+^ T lymphocytes in intestinal lamina propria (LP), and decreased susceptibility to Th1-mediated colitis in mice overexpressing GILZ (17).

Inflammatory bowel diseases such as Crohn’s disease (CD) and ulcerative colitis are chronic and progressive diseases of the gastrointestinal tract. Despite intensive research, our understanding of the pathogenesis of IBDs remains incomplete. T cells are known to play a key role in the pathogenesis of IBDs, and a more intensive Th1 cell response is observed in IBD patients (18, 19).

The role of B cells in IBD is less clear, although they play an important role in controlling mucosal homeostasis in the gut, including antibody (Ab) production, antigen presentation, and co-stimulation of T lymphocytes (20, 21). In addition to their role as conventional Ab-producing B cells, experimental evidence shows that cytokine production by novel subsets of B cells may also affect immune regulatory functions. For instance, IL-10-producing B cells, also called regulatory B (Breg) cells, play an essential role in modulating inflammation and autoimmunity (22). When stimulated, B cells may produce a wide range of cytokines such as IL-4, IL-17, and IFN-γ (23–25), thereby influencing the responses mediated by effector CD4^+^ T cells (26, 27). However, the factors involved in the activation, expansion, and function of cytokine-producing B cells remain poorly characterized.

Recently, we demonstrated an important role of GILZ in B cell survival (28). We showed that lack of GILZ in mice in which B cell homeostasis was perturbed resulted in B cell lymphocytosis (28). In this study, we investigated whether GILZ expression in B cells contributes to the control of inflammatory processes in the gut, such as the production of pro- and/or anti-inflammatory cytokines, and explored whether this alters the severity of colitis in mice. We found that GILZ regulates IFN-γ expression in B cells, and GILZ-deficient B cells produced more IFN-γ, associated with increased AP-1 transcriptional activity. Increased IFN-γ production by B cells lacking GILZ skewed wild-type (WT) CD4^+^ T lymphocytes toward a Th1 phenotype, increased IFN-γ production, and enhanced susceptibility to experimental colitis in mice.

## Materials and Methods

### Mice

Mice bearing a floxed *gilz* allele were generated as described previously (29) and maintained in a C57Bl/6J background. B-conditional *gilz* knock-out (KO) animals (gilz B cKO) were obtained by crossing mice bearing *gilz* flox alleles with transgenic mice bearing the CD19-CRE transgene (30), resulting in the deletion of *gilz* specifically in B cells (Figure S1 in Supplementary Material), as described previously (28). Animal care was in compliance with regulations in Italy (DL 26/2014) and Europe (EU Directive 2010/63/EU).

### Dinitrobenzene Sulfonic Acid (DNBS)-Induced Colitis

To induce colitis, 10- to 14-week-old C57BL/6 male mice were anesthetized with sodium thiopental (30 mg/kg) and xylazine (10 mg/kg). A 2 mg sample of 2,4-DNBS (Sigma-Aldrich) or vehicle (50% ethanol) was administered intrarectally. Body weight, stool consistency, and rectal bleeding were examined daily, and total clinical score was calculated as described previously (17). Disease score was evaluated as described previously (17) according to the following criteria: 0 = no damage, 1 = localized hyperemia without ulcers, 2 = linear ulcers with no significant inflammation, 3 = linear ulcers with inflammation at one site, 4 = two or more major sites of inflammation and ulceration extending greater than 1 cm along the length of the colon. To assess whether GILZ protein administration protected against the development of colitis, mice receiving DNBS were randomized to receive no treatment (DNBS alone) or 0.5 mg/kg TAT–GILZ fusion protein administered intraperitoneally for 3 days, starting on the same day as DNBS rectal administration.

### Blood Collection and Hematocrit

Blood was collected from the retro-orbital plexus of anesthetized mice for hematocrit determination and FC analysis. Blood collection tubes were prepared with 20 µl of 0.5 M EDTA (pH 8). Analyses were performed using a Hemavet 950 (Drew Scientific) calibrated for mouse blood. Parameters determined included white blood cell (WBC) count, neutrophil count, lymphocyte count, monocyte count, eosinophil count, and basophil count.

### Isolation and Culturing of B and T Lymphocytes

B and T cells for *in vitro* assays were isolated from spleen tissue of 10- to 14-week-old WT and gilz B cKO mice using Dynabeads Mouse CD43 Untouched B Cells and a Dynabeads Untouched Mouse CD4 Cell Kit (Thermo Fisher Scientific), respectively, following the manufacturer’s instructions. For *in vitro* cultures, cells were maintained at a density of 2 × 10^6^/ml for 30 min or 5 h at 37°C in fully supplemented RPMI medium, with or without stimulation with 50 ng/ml phorbol 12-myristate 13-acetate (Sigma-Aldrich), 1 µg/ml ionomycin (Sigma-Aldrich), and 10 µg/ml lipopolysaccharide (Sigma-Aldrich).

### LP Cell Isolation

Segments of the colon were incubated for 15 min at 37°C with Dissociation buffer consisting of Ca/Mag-free RPMI containing 5 mM EDTA, 10 mM HEPES, and 1 mM dithiothreitol (DTT), then extensively washed with complete RPMI containing 10 mM HEPES and 10 mM 10% fetal bovine serum (FBS). Fragments were then incubated with pre-warmed Digestion buffer (RPMI complete containing 0.5 mg/ml collagenase and 0.5 mg/ml DNaseI) for 20 min at 37°C. After washing, cells were subjected to density gradient centrifugation in 40/80% Percoll, and LP cells harvested from the interface were washed with phosphate-buffered saline (PBS) containing 2% FBS. Freshly isolated cells were resuspended in PBS for flow cytometric analysis.

### Enzyme-Linked Immunosorbent Assay (ELISA)

Analysis of Abs in sera collected from 12- to 18-month-old mice was performed by ELISA using IL-4, IL-10, IL-17, and IFN-γ ELISA Kits (Alpha Diagnostic International) according to the manufacturer’s instructions.

### Quantitative Real-Time PCR (qPCR)

RNA was isolated using an RNeasy Plus Micro Kit (Qiagen) and reverse-transcribed using a High-Capacity cDNA Reverse Transcription Kit (Applied Biosystems). qPCR was performed using the 7300 Real-Time PCR System (Applied Biosystems) and TaqMan Gene Expression Master Mix (Applied Biosystems). The qPCR TaqMan probes (Applied Biosystems) were as follows: IFN-γ, Mm01168134_m1; IL-4, Mm99999154_m1; IL-10, Mm01288386_m1; IL-17, Mm00439618; Actb, 4352341E.

### Western Blotting

Protein extracts were obtained using RIPA buffer supplemented with protease (Sigma-Aldrich) and phosphatase (Thermo Fisher Scientific) inhibitor cocktails as described previously (31). Separation of nuclear and cytoplasmic fractions was performed using NE-PER Nuclear and Cytoplasmic Extraction Reagents (Thermo Fisher Scientific). Western blot (WB) analyses were performed with Abs recognizing GILZ (clone CFMKG15, eBioscience), c-Jun (clone G-7, Santa Cruz), lamin B1 (rabbit polyclonal, Abcam), or β-tubulin (Sigma-Aldrich) as previously described (31). Densitometric analysis of plots was carried out using ImageJ software (32).

### Abs and Flow Cytometry

Monoclonal antibodies used for flow cytometry analyses and *in vitro* assays are listed in Table S1 in Supplementary Material. Analyses were performed using the ATTUNE NxT three-laser standard configuration (Life Technologies), and data were analyzed using Flow Jo software (Tree Star).

### Chromatin Immunoprecipitation (ChIP) Assay

Chromatin immunoprecipitation assays were performed as described previously (31). In brief, cells were fixed in 1% PFA and sonicated on ice. Pre-cleared lysates were incubated overnight at 4°C with polyclonal anti-c-Jun (Abcam), polyclonal anti-acetyl histone H3 (AcH3, Millipore), or control polyclonal rabbit IgG (Cell Signaling). Immunocomplexes were collected using a Chip Assay Kit (Millipore), and qPCR analysis was performed using Power SYBR Green PCR Master Mix (Applied Biosystems). The following primers were used for chip analysis of the IFN-γ promoter: for the c-Jun binding site, For = GCTGTCTCATCGTCAGAGAGCCCA, Rev = TGATCGAAGGCTCCTCGGGATTACG; for the control region (CR), For = CTTAGGCTGGGAGGTTGTGT, Rev = TTCTCACTCCCTCCTACCCA.

### Statistical Analysis

All statistical analysis was performed with Prism 6.0 (GraphPad). Results shown in figures are representative of at least three independent experiments unless otherwise indicated (*n* = *x* refers to the total number of animals used). The nonparametric Mann–Whitney *U* test or two-tailed unpaired Student’s *t*-test was used for statistical comparison (*p < 0.05; **p < 0.005; ***p < 0.001).

## Results

### B Cell-Specific GILZ KO (Gilz B cKO) Mice Exhibit B Cell Lymphocytosis

**Figure 1:**
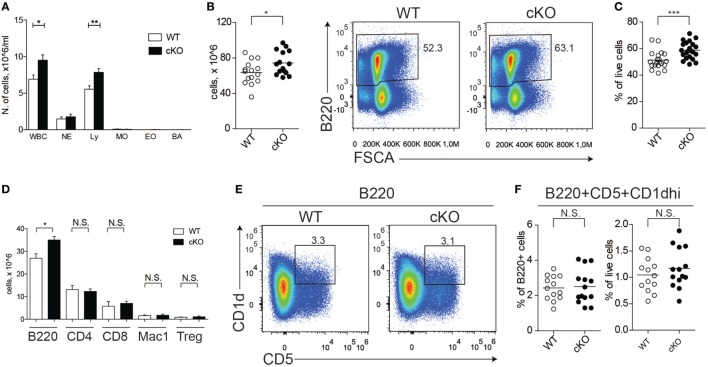
Lack of glucocorticoid-induced leucine zipper (GILZ) in B cells increases B cell abundance, but not for the B220^+^CD5^+^CD1d^hi^ [regulatory B (Breg)] subset. **(A)** Blood cell counts in aged (7- to 8-month olds) wild-type (WT) and gilz B cKO mice; abbreviations in *x*-axis indicate different blood cell population, as follows: WBC, white blood cells; NE, neutrophils; Ly, lymphocytes; MO, monocytes; EO, eosinophils; BA, basophils. **(B)** Total number of cells in the spleen of WT and gilz B cKO mice. **(C)** Flow cytometry analysis of B220^+^ expression in the spleen of WT and gilz B cKO mice. The graph on the right represents the frequency of B220^+^ cells in the spleen. **(D)** Number of leukocyte subsets, reported as B220^+^ B cells (B220), CD4^+^ T cells (CD4), CD8^+^ T cells (CD8), Mac1^+^ Gr1^−^ monocytes/macrophages (Mac1), Cd4^+^FoxP3^+^ regulatory T cells (Tregs), in the spleen of WT and gilz B cKO mice (*n* = 14, 15). **(E)** Flow cytometry analysis of Breg cells in the spleen of WT and gilz B cKO mice. **(F)** Frequency and number of Breg cells in the spleen of WT and gilz B cKO mice (*n* = 12). N.S., not significant; statistical analysis was performed using the unpaired Student’s *t*-test (*p < 0.05, **p < 0.005, ***p < 0.001).

We previously reported that GILZ is expressed in B cells and regulates spontaneous apoptosis, thereby contributing to the control of B cell homeostasis (28). To confirm the cell-intrinsic role of GILZ in B cells, we crossed mice bearing the floxed GILZ allele with mice in which Cre recombinase is expressed under the control of the CD19 promoter (30), thus achieving a B cell-restricted GILZ deletion (hereafter referred to as gilz B cKO mice; Figure S1 in Supplementary Material). As observed for mice with GILZ deletion in all tissues (28), total WBC and lymphocyte counts were increased in the peripheral blood of gilz B cKO mice compared with WT mice (Figure 1A). Spleen tissue from gilz B cKO mice also displayed increased cellularity (Figure 1B), associated with increased frequency of B220^+^ cells compared with WT mice (Figure 1C). The number of B cells was significantly higher in the spleens of gilz B cKO mice compared with WT mice, while the number of blood cells of other myeloid and lymphoid lineage, including CD4^+^, CD8^+^, and Mac-1^+^, remained constant. Similarly, no difference was found in CD4^+^ regulatory T (Treg) cell frequency between WT and gilz B cKO mice (Figure 1D, last two columns on the right). These results demonstrate that the increase in the number of B cells following B cell-specific GILZ deletion in gilz B cKO mice is cell-intrinsic to the B cell population.

We next evaluated whether the absence of GILZ alters the frequency of B cell subsets within lymphoid tissues, including Breg cells. Flow cytometry analysis of splenic B cells revealed a similar frequency and number of Breg cells in WT and gilz B cKO mice (Figures 1E,F). Moreover, GILZ-deficient mice did not exhibit altered expression of Breg effector cytokines IL-10 or TGF-β in serum (Figure S2A in Supplementary Material) or in the supernatants of B cells cultured *in vitro* (Figure S2B in Supplementary Material), suggesting that Breg cell function was not perturbed in gilz cKO mice. Other B cell subsets, including follicular, marginal, and recirculating B cells, were also present in similar frequencies in spleen tissue and peripheral blood of WT and gilz B cKO mice (Figure S3A in Supplementary Material).

In addition, we did not detect any difference in the frequency or number of plasma cells (defined as B220^+^IgD^lo^CD138^hi^; Figure S3B in Supplementary Material) or serum levels of immunoglobulins (Figure S3C in Supplementary Material) in WT and gilz B cKO mice. Furthermore, we did not detect differences in the activation status of B cells isolated from spleens of WT and gilz B cKO mice, as measured by the expression of activation markers CD80 and CD86 (Figure S4 in Supplementary Material). Together, these results suggest that the relative composition of B cell subsets was not perturbed by the absence of GILZ.

### Lack of GILZ in B Cells Results in Increased IFN-γ Production

**Figure 2:**
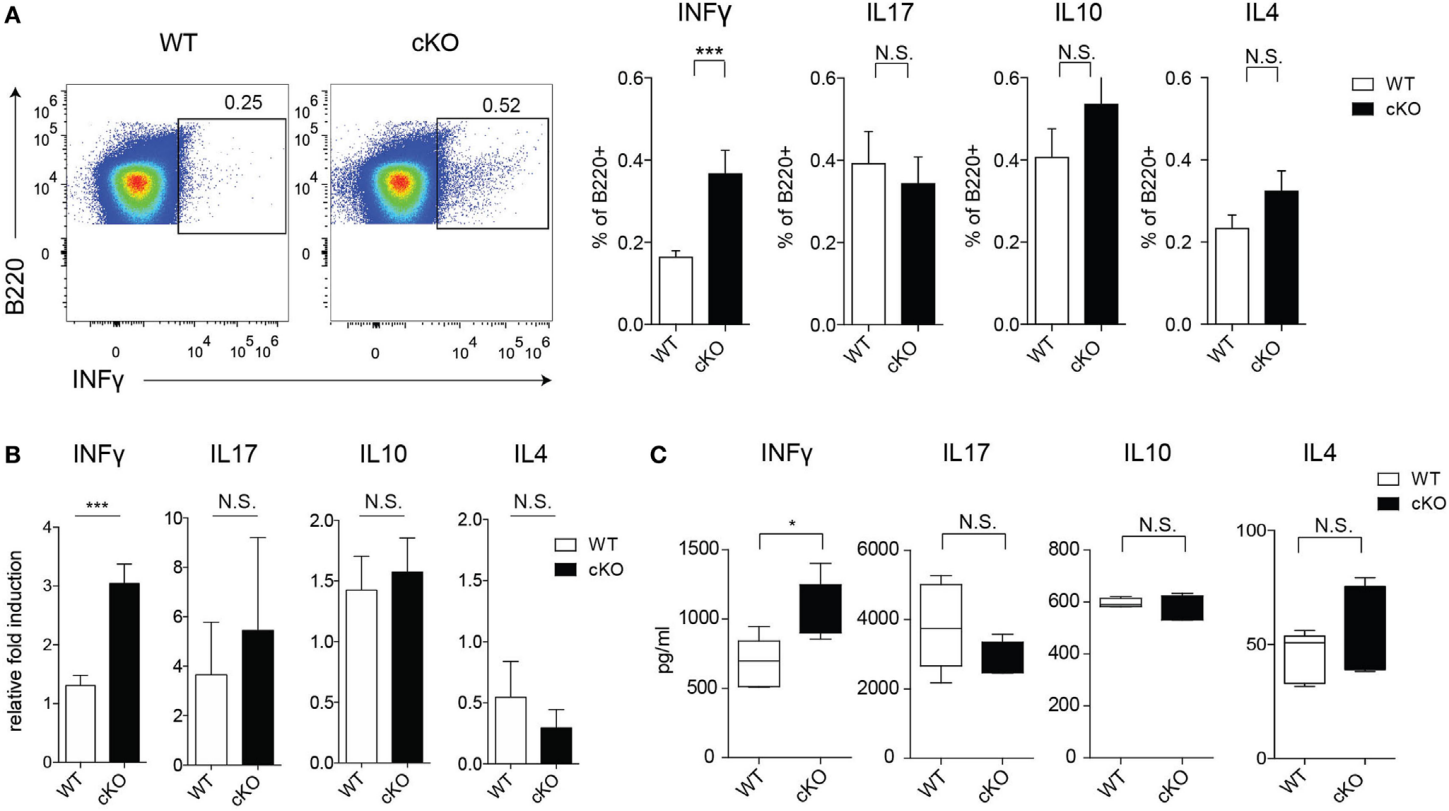
Lack of glucocorticoid-induced leucine zipper (GILZ) in B cells leads to increased production of IFN-γ in splenic B cells. **(A)** Flow cytometry analysis of IFN-γ, interleukin (IL)-17, IL-10, and IL-4 *ex vivo* production by B220^+^ cells in the spleen of wild-type (WT) and gilz B cKO mice. Plots in the left panels are representative of flow cytometry analysis of IFN-γ production by B220^+^ cells. **(B)** Quantitative real-time PCR analysis of cytokine produced by B220^+^ cells purified from spleen tissue stimulated *in vitro* with phorbol 12-myristate 13-acetate/ionomycin and lipopolysaccharide for 24 h. Data are derived from three independent experiments. **(C)** Concentration of IFN-γ, IL-17, IL-10, and IL-4 measured by enzyme-linked immunosorbent assay in sera of 9- to 12-month-old WT and gilz B cKO mice (*n* = 7, 7). N.S., not significant; statistical analysis was performed using the unpaired Student’s *t*-test (*p < 0.05, ***p < 0.001).

To assess the possible role of GILZ in Ab-independent functions of B cells, we evaluated cytokine production by splenic B cells in mice under physiological conditions. Figures 2A,B shows that lack of GILZ in B cells resulted in increased production of IFN-γ by B cells, but not other cytokines such as IL-4, IL-10, and IL-17. Consistently, increased IFN-γ production was detected in sera from aged gilz B cKO cells compared with WT mice, while expression of IL-17, IL-10, and IL-4 was not significantly different (Figure 2C). These results suggest that GILZ is important in restraining the expression of IFN-γ in B cells, and lack of GILZ results in the elevated systemic presence of IFN-γ in gilz B cKO mice.

### Lack of GILZ in B Cells Stimulates IFN-γ Production in WT CD4^+^ T Cells

**Figure 3:**
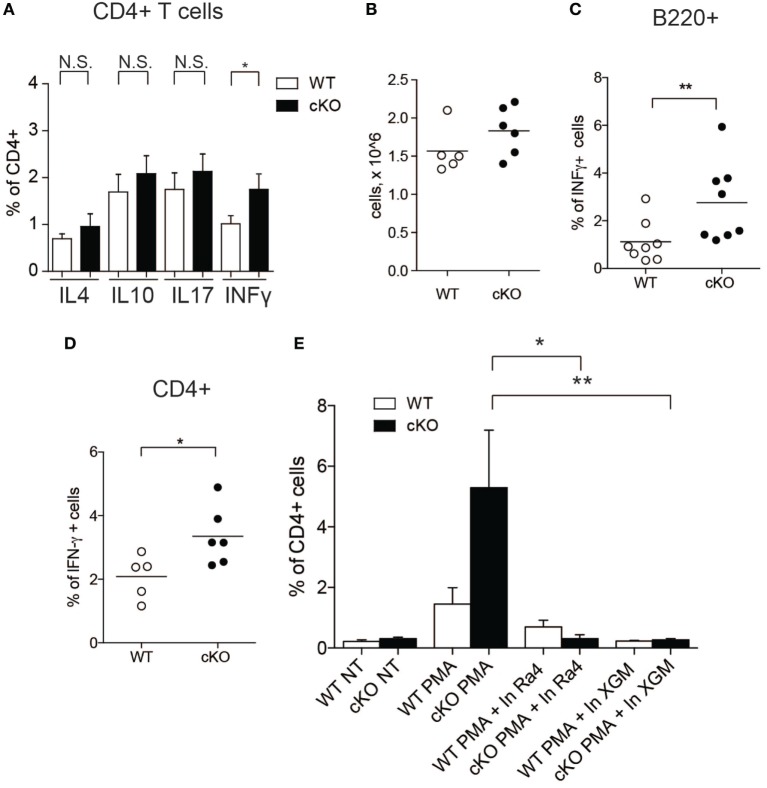
Lack of glucocorticoid-induced leucine zipper (GILZ) in B cells leads to increased production of IFN-γ by splenic T cells and in peripheral blood. **(A)** Analysis of IFN-γ cytokine production by CD4^+^ cells in the spleen of wild-type (WT) and gilz B cKO mice. **(B)** Cell counts of WT, gilz B cKO B, and T cells co-cultured and stimulated *in vitro* with phorbol 12-myristate 13-acetate (PMA)/ionomycin and lipopolysaccharide (LPS) for 24 h. **(C,D)** Flow cytometry analysis of IFN-γ production in B cells [**(C)**, B220^+^] or T cells [**(D)**, CD4^+^] co-cultured and stimulated *in vitro* with PMA/ionomycin and LPS for 24 h. **(E)** Flow cytometry analysis of IFN-γ production in B and T cells co-cultured and stimulated *in vitro* with PMA/ionomycin and LPS for 24 h with or without anti-IFN-γ antibodies (Ra4 and XGM; *n* = 9, 10); N.S., not significant; statistical analysis was performed using the unpaired Student’s *t*-test (*p < 0.05, **p < 0.005).

Since IFN-γ influences the function of CD4^+^ T cells (33), we tested whether increased IFN-γ production by GILZ-deficient B cells results in altered T cell activity in gilz B cKO mice. We measured the expression of various cytokines in splenic CD4^+^ T cells in WT and gilz B cKO mice by flow cytometry. Figure 3A shows that expression of IFN-γ was significantly increased in T cells isolated from spleens of gilz B cKO mice compared with WT mice, while expression of IL-4, IL-10, and IL-17 did not differ significantly. To test whether increased IFN-γ expression in WT T cells results from the co-presence of GILZ-deficient B cells producing IFN-γ, we co-cultured T cells purified from WT mice with B cells purified from gilz B cKO mice, but this did not significantly alter the total number of cultured cells *in vitro* (Figure 3B). Flow cytometry analysis of cytokine production showed that co-culturing with IFN-γ-producing GILZ-deficient B cells (Figure 3C) significantly increased the expression of IFN-γ in WT CD4^+^ T cells (Figure 3D) without affecting the expression of other cytokines such as IL-17 and IL-10 (Figure S5 in Supplementary Material). Importantly, addition of anti-IFN-γ Abs to cell cultures (34, 35) counteracted the increased IFN-γ production by WT T cells co-cultured with gilz B cKO B cells (Figure 3E). These results demonstrate that IFN-γ produced by GILZ-deficient B cells stimulates IFN-γ production in T cells.

### GILZ Inhibits AP-1-Dependent IFN-γ Production

**Figure 4:**
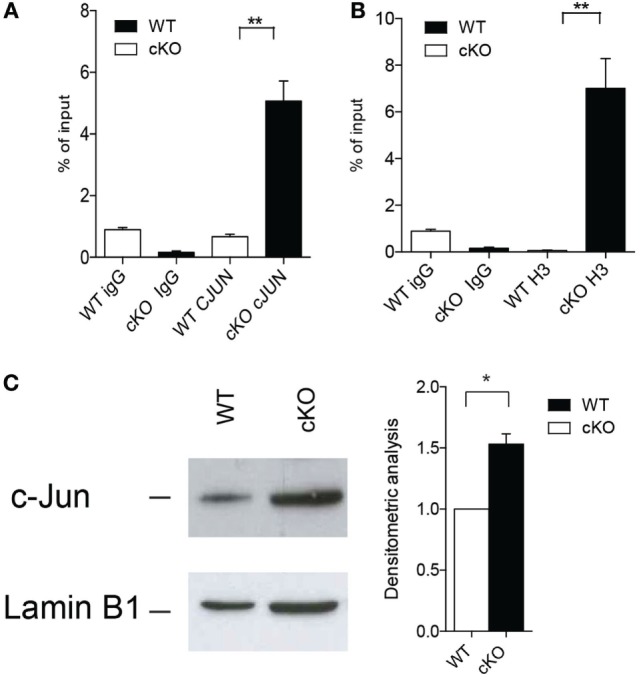
Lack of glucocorticoid-induced leucine zipper (GILZ) in B cells increases the production of IFN-γ *via* enhanced AP-1 transcriptional activity. **(A,B)** Chromatin immunoprecipitation analysis of the *ifng* promoter in spleen cells of wild-type (WT) and gilz B cKO mice with anti-c-Jun antibody **(A)** or anti-acetylated histone H3 [**(B)**; *n* = 9]. Statistical analysis was performed using the unpaired Student’s *t*-test (**p < 0.005). **(C)** Western blot analysis of nuclear c-Jun levels in purified B cells from spleens of WT and gilz B cKO mice. The graph on the right shows the results of densitometry analysis of c-Jun relative to the housekeeping gene lamin B1. Statistical analysis was performed using the nonparametric Mann–Whitney *U* test (*p < 0.05).

Activation of the transcription factor AP-1 is important for the modulation of IFN-γ production by effector Th1 and by B cells (36, 37), and GILZ binds directly to AP-1 and represses its transcriptional activity in T lymphocytes (12). We found that c-Jun, an integral part of the AP-1 transcription factor, binds more extensively to the IFN-γ promoter in purified B cells lacking GILZ (Figure 4A) compare with WT B cells, as revealed by ChIP in B cells purified from spleens of WT or gilz B cKO animals. Consequently, the IFN-γ promoter is more transcriptionally active, as indicated by the enhanced acetylation of histone H3 (AcH3) in gilz B cKO B cells compared with WT B cells (Figure 4B). This was associated with the enhanced nuclear translocation of c-Jun in GILZ-deficient B cells compared with WT B cells, as assessed by WB analysis of c-Jun levels in nuclear cell fractions (Figure 4C). These results suggest that GILZ restrains IFN-γ production by inhibiting nuclear translocation of AP-1, and thus its transcriptional activity in B cells.

### DNBS-Induced Colitis Is More Severe in Gilz B Cell-Deficient cKO Mice

**Figure 5:**
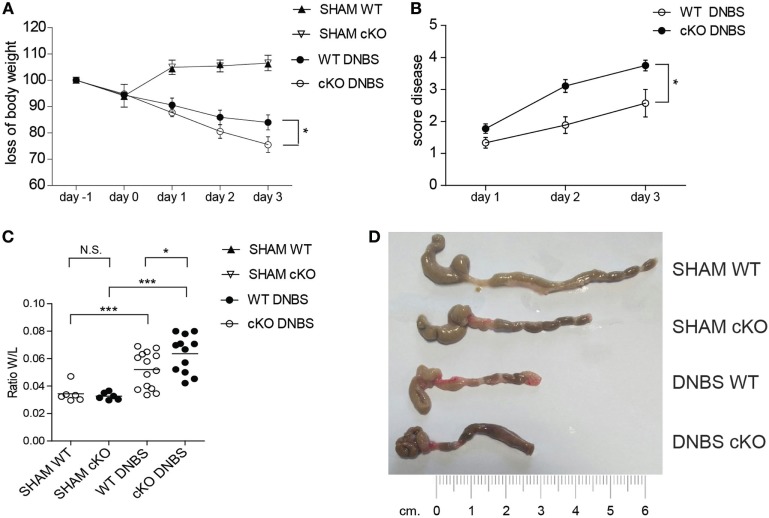
Lack of glucocorticoid-induced leucine zipper (GILZ) exacerbates 2,4-dinitrobenzene sulfonic acid (DNBS)-induced colitis. **(A)** Loss of body weight in wild-type (WT) and gilz B cKO mice treated intrarectally with vehicle (Sham) or DNBS. **(B)** Daily measurement of total disease score in WT and gilz B cKO mice during DNBS-induced colitis. **(C)** Colon weight/length ratio of WT and gilz B cKO mice intrarectally administered with vehicle (Sham) or DNBS. **(D)** Representative macroscopic images of the colon of WT and gilz B cKO mice intrarectally administered with vehicle (Sham) or DNBS are shown (*n* = 9). Statistical analysis was performed using the unpaired Student’s *t*-test (*p < 0.05, ***p < 0.001).

While the pathogenic role of T cells in IBD is well established (38, 39), the role of B cells in IBD is less clear, and the mechanisms involved in the interplay between B and T cells in controlling inflammatory responses in the gut are not well defined. The above results suggest that GILZ-deficient B cells drive T cells toward the Th1 phenotype. Th1 cells are implicated in IBDs and the role of B cells in IBDs has been highlighted in various mouse models (40, 41). To assess whether the loss of GILZ in B cells influences the development of colitis, we used the DNBS-induced colitis Th1-type disease mouse model. We found that lack of GILZ in B cells exacerbates DNBS-induced colitis in gilz cKO mice, as evidenced by a significantly increased body weight loss (Figure 5A) and an elevated disease index score in gilz cKO mice (Figure 5B). Differences in colon weight/length ratio, a marker of tissue edema (Figure 5C), obvious reddening and shortening of the colon (Figure 5D), and disrupted colon tissue architecture (Figure S6 in Supplementary Material), were evident in gilz cKO mice compared with WT controls upon induction of colitis, providing further evidence of increased inflammation in experimental colitis upon GILZ deletion in mice.

**Figure 6:**
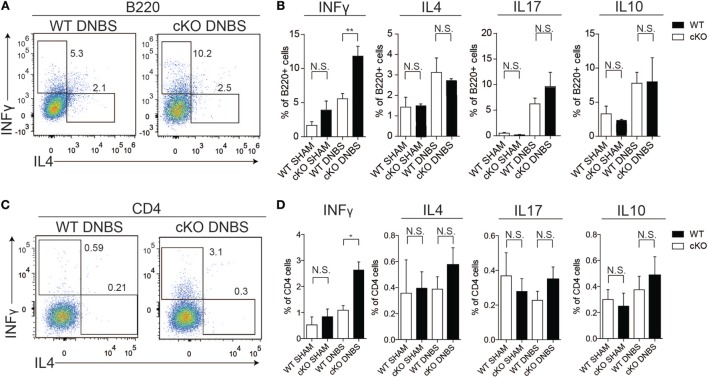
Increased IFN-γ production in lamina propria (LP) of the colon in gilz B cKO mice upon dinitrobenzene sulfonic acid (DNBS)-induced colitis. **(A)** Representative plots of flow cytometry analysis of IFN-γ and IL4 production by B220^+^ cells isolated from LP of the colon of wild-type (WT) and gilz B cKO mice upon DNBS-induced colitis. **(B)** Graphs representing IFN-γ, interleukin (IL)-4, IL-17, and IL-10 production by B220^+^ cells isolated from LP of the colon of WT and gilz B cKO mice following DNBS-induced colitis. **(C)** Representative plots of flow cytometry analysis of IFN-γ and IL4 production by CD4^+^ cells of the colon of WT and cKO mice upon DNBS-induced colitis. **(D)** Graphs representing the frequency of IFN-γ, IL-4, IL-17, and IL-10 production by CD4^+^ cells isolated from LP of the colon of WT and gilz B cKO mice following DNBS-induced colitis (*n* = 10, 9) N.S., not significant, statistical analysis was performed using the unpaired Student’s *t*-test (*p < 0.05, **p < 0.005).

To probe the basis of the increased disease susceptibility in gilz cKO mice, we compared cytokine production by cells isolated from the colon LP of WT and gilz cKO mice treated with DNBS. Similar to the spleen tissue results, we observed increased IFN-γ production by B cells in colon LP of gilz cKO compared with WT controls following administration of DNBS (Figures 6A,B). We also detected increased IFN-γ production by T cells in the LP of gilz cKO animals (Figures 6C,D). Together, these results suggest that increased IFN-γ production in the colon LP is associated with augmented susceptibility to experimental colitis in gilz cKO mice.

### TAT–GILZ Restores Normal IFN-γ Production and Protects Against DNBS-Induced Colitis

**Figure 7:**
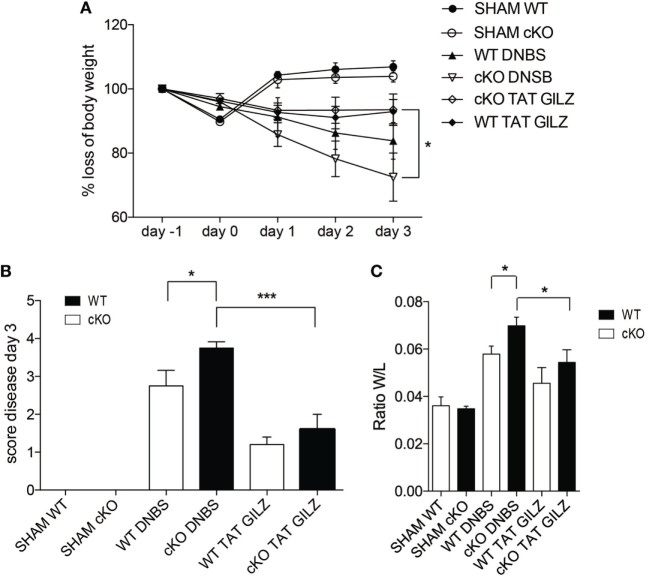
The TAT–glucocorticoid-induced leucine zipper (GILZ) fusion protein reverses the symptoms of dinitrobenzene sulfonic acid (DNBS)-induced colitis. **(A)** Loss of body weight in wild-type (WT) and gilz B cKO mice treated intrarectally with vehicle (Sham) or DNBS, with and without administration of TAT–GILZ fusion protein (daily i.p. administration of 0.5 mg/kg, starting from day 0). **(B)** Daily measurement of total disease score in WT and gilz B cKO mice during DNBS-induced colitis, treated with or without TAT–GILZ fusion protein. **(C)** Colon weight/length ratio of WT and gilz B cKO mice intrarectally administered with vehicle (Sham) or DNBS, treated with and without TAT–GILZ fusion protein. Data are from two independent experiments (*n* = 6, 7). N.S., not significant, statistical analysis was performed using the unpaired Student’s *t*-test (*p < 0.05, **p < 0.005).

The above data indicate that GILZ is required for controlling the responses to inflammatory processes in the gut. To test whether exogenous GILZ administration can reverse colitis symptoms, we treated both WT and gilz cKO mice with recombinant TAT–GILZ fusion protein and evaluated DNBS-induced disease development. We previously demonstrated that recombinant TAT–GILZ can enter cells using the Hiv-TAT sequence that delivers the fusion protein into cells, in this case GILZ linked to the TAT transduction domain (9, 17). As shown in Figure 7, the intra-peritoneal delivery of TAT–GILZ protein protected against DNBS-induced colitis in mice, as evidenced by diminished body weight loss in WT and gilz cKO mice treated with TAT–GILZ compared with non-treated controls (Figure 7A). As expected, disease score (Figure 7B) and colon weight/length ratio (Figure 7C) indicated that treatment with TAT–GILZ decreased disease severity in WT mice (Figures 7B,C, column 5 vs. 3). Interestingly, TAT–GILZ treatment also counteracted the inflammation induced by DNBS in gilz cKO mice (Figures 7B,C, column 6 vs. 4), suggesting that recombinant TAT–GILZ administration had an anti-inflammatory effect and reversed inflammatory disease predisposition in gilz cKO mice.

## Discussion

Despite recent advances in IBD therapy, an ideal treatment remains elusive, and therapy must often be discontinued due to serious side effects. For example, GCs are highly efficacious in therapeutic approaches to IBD, but prolonged treatment causes severe adverse effects that necessitate therapy interruption (1, 2).

The Ab-independent pathogenic role of B cells is emerging as a key player in the development of chronic inflammatory and autoimmune diseases. This immunomodulatory function of B cells is associated with their ability to produce anti- or pro-inflammatory cytokines. Dysfunction of specific B cell subsets has been linked to the development or exacerbation of various diseases, including IBDs (42, 43). A decrease in the abundance of B cells producing IL-10 (Breg cells) has been observed in patients with CD (44), and adoptive transfer of IL-10-producing B cells ameliorates intestinal inflammation in a mouse model of colitis (44). B cells can also produce pro-inflammatory cytokines, and perturbation of cytokine-producing B cell subsets has been linked to the pathology of inflammatory and autoimmune diseases (20, 22, 25, 36). However, the mechanisms regulating the production of immuno-regulatory cytokines by B cells, and their role in the immunopathology of IBDs, remain undefined.

T and B cells are essential regulators of adaptive immune responses, and a growing body of evidence suggests that B cells possess immunomodulatory functions due to the production of specific cytokines and their interactions with T cell subpopulations. Through cytokine production, B cells promote the differentiation and maintenance of Treg cells and differentiation of other T cell subsets, including Th1, Th2, and Th17 (26, 45, 46). Importantly, B cells can stimulate Th1-type immunity by producing IFN-γ, as demonstrated in *Salmonella typhimurium* infection experiments (47).

In this study, we aimed to address the role GILZ, which is induced by GCs, in B cells using a B cell conditional gilz KO (B cKO) mouse model. We previously demonstrated that lack of GILZ expression modulates B cell homeostasis (28). Herein, we showed that restriction of *gilz* deletion to B cells recapitulates the B cell lymphocytosis phenotype, as demonstrated by the increased number of B220^+^ cells in the spleen and peripheral blood of gilz B cKO mice compared with WT controls. Moreover, gilz B cKO mice exhibited increased susceptibility to experimental colitis. The regulatory activities of a subset of B cells, namely Breg cells, have been linked to the modulation of inflammation and related diseases (42, 44). Analysis of B cells in WT and gilz B cKO mice revealed that ablation of *gilz* expression in B cells does not perturb the frequency of CD1^hi^CD5^+^ Breg cells, or production of their regulatory cytokines such as IL-10 and TGF-β. Similarly, *gilz* deficiency in B cells did not affect the production of Treg cells, even though lack of *gilz* specifically in T cells results in impaired generation of Treg cells (31). These observations indicate that deregulation of Breg and Treg functions, both shown to be important modulators of inflammatory diseases including IBDs, is not the mechanism responsible for increased inflammation in gilz B cKO mice.

B cells can act as accessory cells that regulate T cell differentiation and cytokine production (40, 41). In this study, we observed that lack of GILZ in B cells led to increased production of IFN-γ in splenic B cells, but not of other cytokines such as IL-4 or IL-17. Increased IFN-γ levels were also detected in sera of aged gilz B cKO mice compared with WT mice, suggesting an increase in IFN-γ may be relevant to inflammatory processes, although no other clear signs of inflammation at steady state were observed in aged mice. Interestingly, we found that T cells also contributed to the increased production of IFN-γ in gilz B cKO mice, and *in vitro* co-culture experiments confirm that WT T cells were stimulated to produce IFN-γ by *gilz*-deficient B cells. To further demonstrate that GILZ-deficient IFN-γ-producing B cells influenced the production of IFN-γ by WT T cells, we used two different anti-IFN-γ blocking Abs in an attempt to reverse the hyper-production of IFN-γ by T cells co-cultured with gilz B cKO B cells. The observed pharmacological rescue of normal IFN-γ production by the anti-IFN-γ Abs indicates that GILZ controls CD4^+^ T cell activity *via* B cell production of IFN-γ. These results suggest that GILZ influences the fine-tuning of B/T cell interactions, with consequent regulation of adaptive immune responses, such as the production of the pro-inflammatory cytokine IFN-γ by both B and T lymphocytes.

Glucocorticoid-induced leucine zipper is a leucine zipper protein that physically interacts with and modulates the functions of several other molecules (8, 10, 12–14, 29, 31). Various transcription factors regulate the activity of the *ifng* promoter, as revealed by bioinformatic analysis and previous studies (48). Among these, the AP-1 transcription factor plays an essential role in *ifng* transcriptional regulation, and at least two canonical AP-1 binding sites are present in the *ifng* proximal promoter. GILZ is able to interact with and inhibit the activity of AP-1 (12). We therefore assessed the activity of the *ifng* promoter and detected an increased AP-1 presence and enhanced acetylation of histone H3 in the proximity of the AP-1 binding site in GILZ-deficient B cells compared with WT B cells, suggesting that the enhanced activity of the *ifng* promoter in GILZ-deficient B cells is mediated by the AP-1 transcription factor. These results indicate that GILZ regulates AP-1 binding to the IFN-γ promoter, and hence its transcriptional activity.

To demonstrate that lack of GILZ in B cells affects functional interactions between B and T cells during inflammatory processes *in vivo*, we used DNBS-induced Th1-type experimental colitis model and monitored signs of disease development in WT and gilz B cKO mice. The results clearly showed that lack of GILZ was associated with exacerbated DNBS-induced colitis, demonstrating that GILZ acts as an anti-inflammatory protein in this cellular context, and that its activity in B cells is important for restraining pathological inflammation of the colon. Interestingly, we found that colitis in mice was antagonized by the *in vivo* administration of recombinant GILZ protein, in both WT and gilz B cKO mice, consistent with previous findings in other experimental mouse models of inflammatory diseases (9, 17, 49).

Increased leukocyte infiltration into the gut, and elevated production of inflammatory cytokines such as IFN-γ, are characteristic features of IBDs in humans and other animals (50). We detected an increase in IFN-γ production in cells isolated from colon LP of gilz B cKO mice following DNBS-induced colitis. Interestingly, we confirmed that T cells also contribute to the increased production of IFN-γ in colon LP in gilz B cKO mice in response to DNBS treatment, suggesting that the aberrant function of B cells determined by GILZ deletion enhances the pro-inflammatory activity of B and T cells in the colon during disease development. These findings point to an important Ab-independent function of B cells that modulate T cell responses in inflammatory diseases. Given that increased IFN-γ production in T cells is observed not only in colon LP but also in spleen tissue, we cannot exclude the possibility that T cells are primed by B cells in peripheral lymphoid organs, leading to their enhanced pro-inflammatory activity in LP. Further studies aimed at dissecting the mechanisms of potential cross talk between B and T cells in disease development are therefore warranted.

In conclusion, the results of this study indicate that GILZ regulates IFN-γ production in B cells by controlling AP-1 transcriptional activity, and modulation of GILZ expression is important for regulating B and T cell activity and the development of inflammatory diseases of the GI tract, such as colitis. Therefore, GILZ could represent a novel target for therapies aimed at treating inflammatory diseases such as IBDs.

## Ethics Statement

The Italian Ministry of Health, Direzione Generale della Sanità Animale e dei Farmaci veterinari, approved the animal use in the proposed research (Approval number 251/2015-PR). The procedures related to animal use are accurately described in the proposal and are conform to all regulations protecting animals used for research purposes, including those of the Italian DL 26/2014 and in Directive 2010/63/EU of the European Parliament and of the Council of 22 September 2010 on the protection of animals used for scientific purposes.

## Author Contributions

SB participated to study concept and design and supervision; he designed and performed the experiments and analyzed the data. He wrote the manuscript. He obtained funding to support the project leading to this publication. DS, SF, and AG performed the experiments and analyzed the data. FA performed the experiments and statistical analysis of the data. SR performed the experiments and participated to critical revision of the manuscript for important intellectual content. GM participates to the drafting of the manuscript and to critical revision of the manuscript for important intellectual content. She obtained funding to support the project leading to this publication. OB performed the experiments and analyzed the data. She participates to the drafting of the manuscript and to critical revision of the manuscript for important intellectual content. She obtained funding to support the project leading to this publication. CR made substantial contributions to study concept and design and supervision; he also participates in drafting the article or revising it critically for important intellectual content. He obtained funding to support the project leading to this publication.

## Conflict of Interest Statement

The authors declare that the research was conducted in the absence of any commercial or financial relationships that could be construed as a potential conflict of interest.
