# Supplementary material for: Glucocorticoid-Induced Leucine Zipper Inhibits Interferon-Gamma Production in B Cells and Suppresses Colitis in Mice
Source: Front Immunol. 2018 Jul 23;9:1720. doi: 10.3389/fimmu.2018.01720 (PMC6064738; doi:10.3389/fimmu.2018.01720)
Supplement: Supplementary file 1 [file data_sheet_1.PDF]

Supplemental Materials and Methods

ELISA

For analysis of antibodies production by ELISA, sera were collected from Wt and *gilz* cKO mice. ELISA were performed using, IgE, IgM, IgG1 and IgG2 ELISA KIT (Alpha Diagnostic International) according to the manufacturer's instructions.

Histology

Tissues were fixed for 24 h in paraformaldehyde (PFA) solution (4% in PBS 0.1 M) at room temperature, dehydrated by graded ethanol and embedded in Paraplast (Sherwood Medical, Mahwah, NJ). Tissue sections (thickness, 5 μm) were deparaffinized with xylene, stained with hematoxylin & eosin, and studied using light microscopy (LEICA DM 2000 combined with a LEICA ICC50 HD camera).

Flow cytometry analysis of B cell subsets

Analyses were performed using the ATTUNE NxT three-laser standard configuration (Life Technologies) and data were analysed using FlowJo software (TreeStar). Monoclonal antibodies (mAbs) used for flow cytometry analyses were the following:

CD45R (B220) Alexafluor700 anti-human/ mouse clone RA3-6B2 (eBioscience)
CD21/35 APC efluor780 Anti-Mouse clone ebio8D9 (eBioscience)
IgD PE-CY7 Anti-Mouse clone11-26c (eBioscience)
IgM FITC Anti-Mouse clone eB121-15F9 (eBioscience)
CD23 PercP-eFluor710 Anti-Mouse clone B3B4 (eBioscience)
CD138 PE Anti-Mouse clone 281-2 (Biolegend)
CD45R (B220) APC anti-human/mouse clone RA3-6B2 (eBioscience)
