# Supplementary material for: Glucocorticoid-Induced Leucine Zipper Inhibits Interferon-Gamma Production in B Cells and Suppresses Colitis in Mice
Source: Front Immunol. 2018 Jul 23;9:1720. doi: 10.3389/fimmu.2018.01720 (PMC6064738; doi:10.3389/fimmu.2018.01720)
Supplement: Supplementary file 2 [file table_1.PDF]

Table S1. List of Antibodies used in flow cytometry analysis

Antibody	Conjugate	Species	Clone	Supplier	Dilution
CD45R (B220)	Alexafluor700	Anti-Human/ Mouse	RA3-6B2	eBioscience	1:200
CD23	PercP-eFluor710	Anti-Mouse	B3B4	eBioscience	1:200
CD21/35	APC-eFluor780	Anti-Mouse	eBio8D9	eBioscience	1:100
IgD	PE-CY7	Anti-Mouse	11-26	eBioscience	1:200
IgM	FITC	Anti-Mouse	eB121-15F9	eBioscience	1:200
CD1d	PerCP-eFluor710	Anti-Mouse	1B1	eBioscience	1:200
CD5	AF780	Anti-Mouse	53-7.3	eBioscience	1:200
IL-10	PE	Anti-Mouse	JESS-16E3	eBioscience	1:200
IL-17	FITC	Anti-Mouse	eBio17B7	eBioscience	1:200
CD80	APC	Anti-Mouse	16-10A1	eBioscience	1:200
FoxP3	PERCP5.5	Anti-Mouse/Rat	FJK-16s	eBioscience	1:400
CD62L	PECY7	Anti-Mouse	MEL-14	eBioscience	1:200
CD8	PE	Anti-Human/ Mouse	53-6.7	eBioscience	1:800
CD4	APC	Anti-Mouse	GK1.5	eBioscience	1:800
CD86	PE	Anti-Human/ Mouse	GL1	eBioscience	1:200
IL-4	PE-CY7	Anti-Human/ Mouse	BVD6-24G2	eBioscience	1:200
IFN-γ	APC	Anti-Mouse	XMG1.2	eBioscience	1:200
