# Supplementary figures and images for: Glucocorticoid-Induced Leucine Zipper Inhibits Interferon-Gamma Production in B Cells and Suppresses Colitis in Mice
Source: Front Immunol. 2018 Jul 23;9:1720. doi: 10.3389/fimmu.2018.01720 (PMC6064738; doi:10.3389/fimmu.2018.01720)

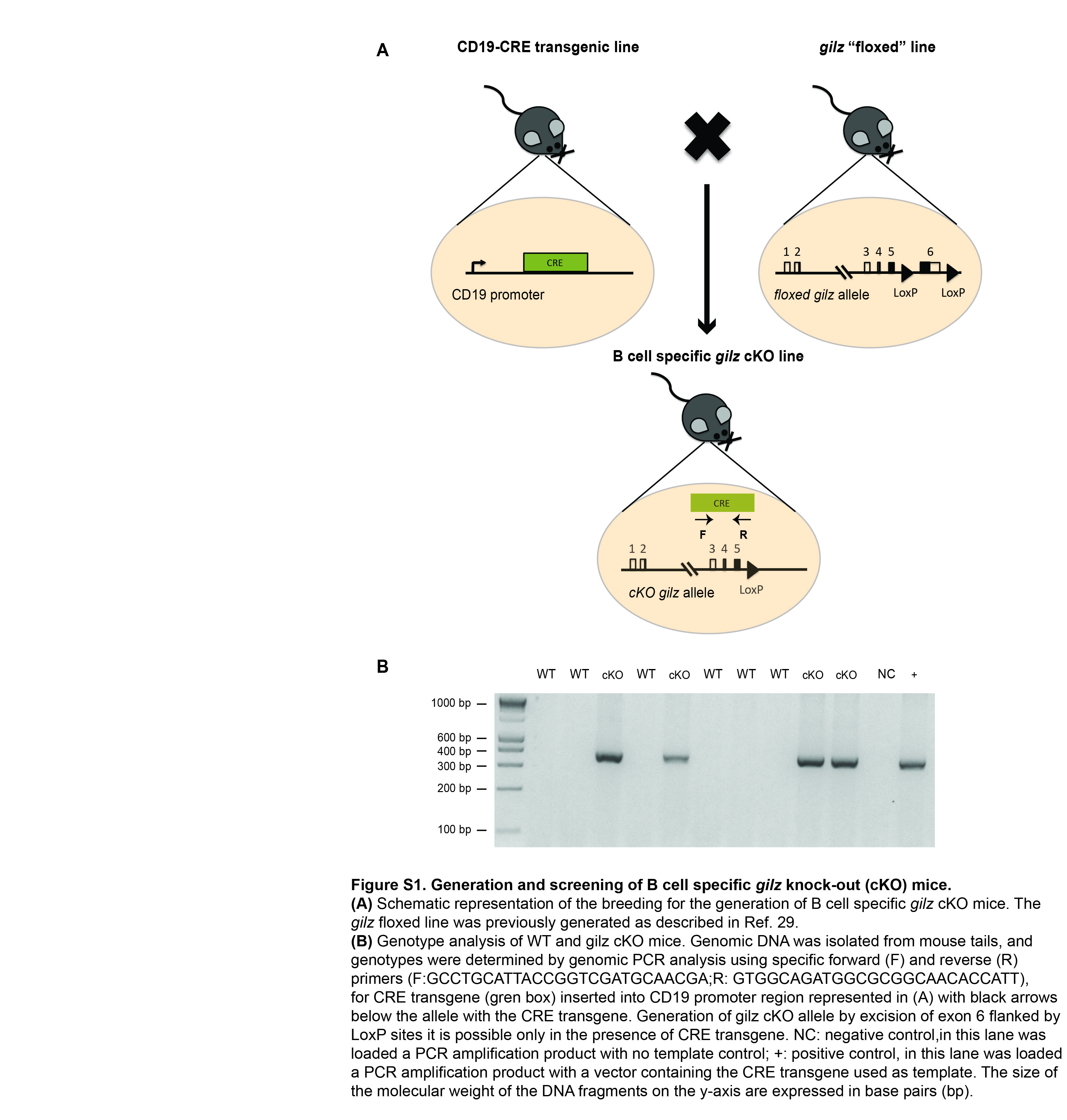

Supplement: Supplementary file 3 [file image_1.jpeg]

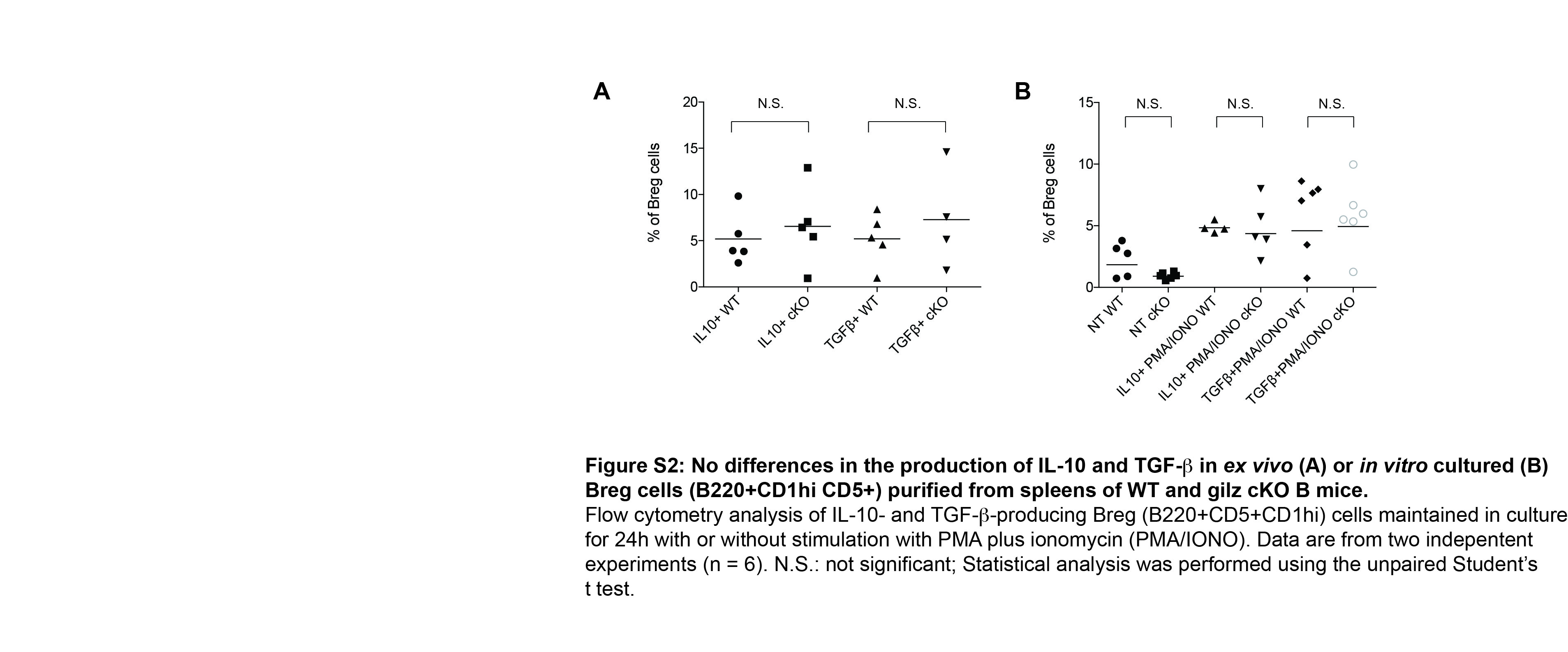

Supplement: Supplementary file 4 [file image_2.jpeg]

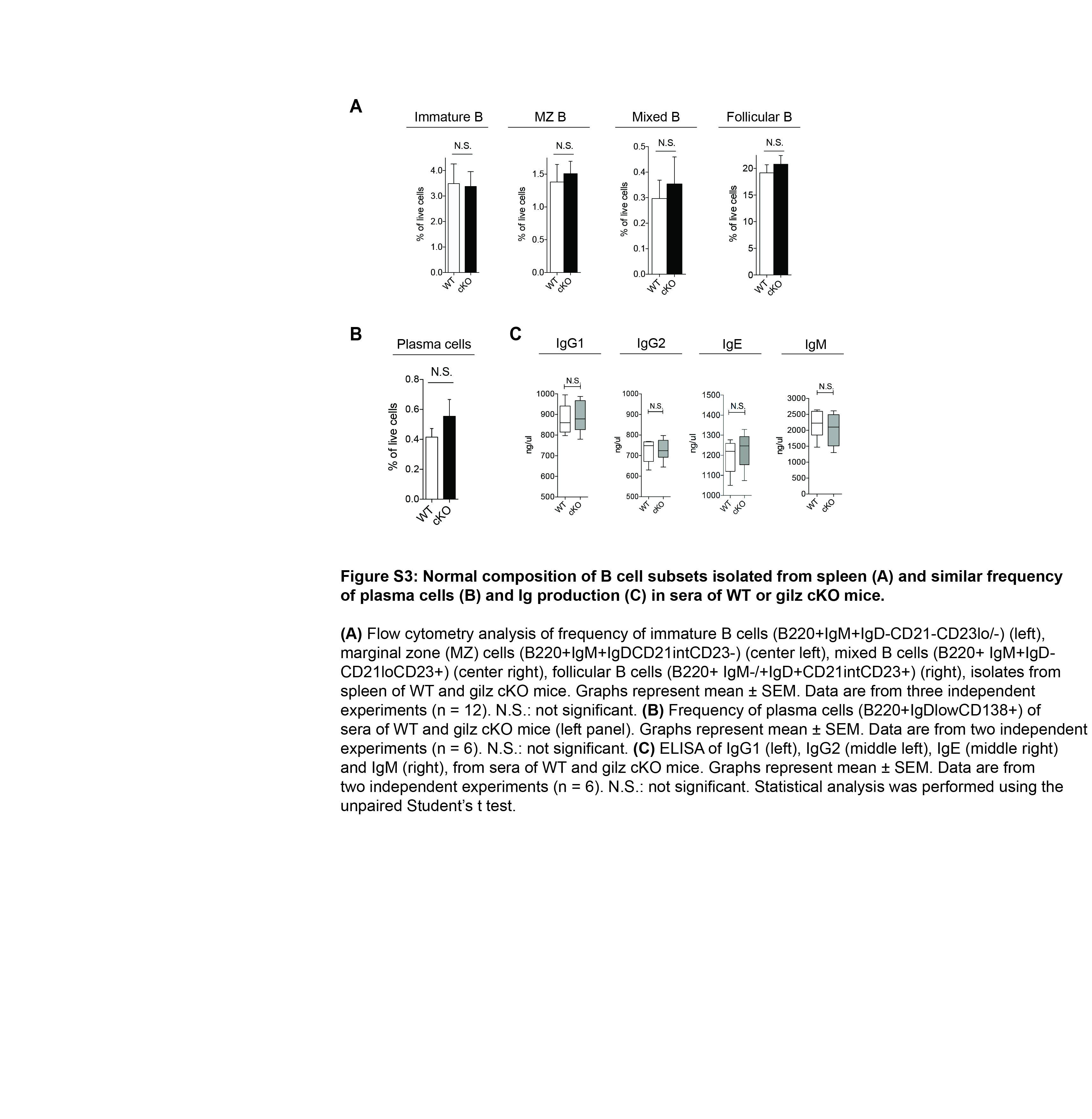

Supplement: Supplementary file 5 [file image_3.jpeg]

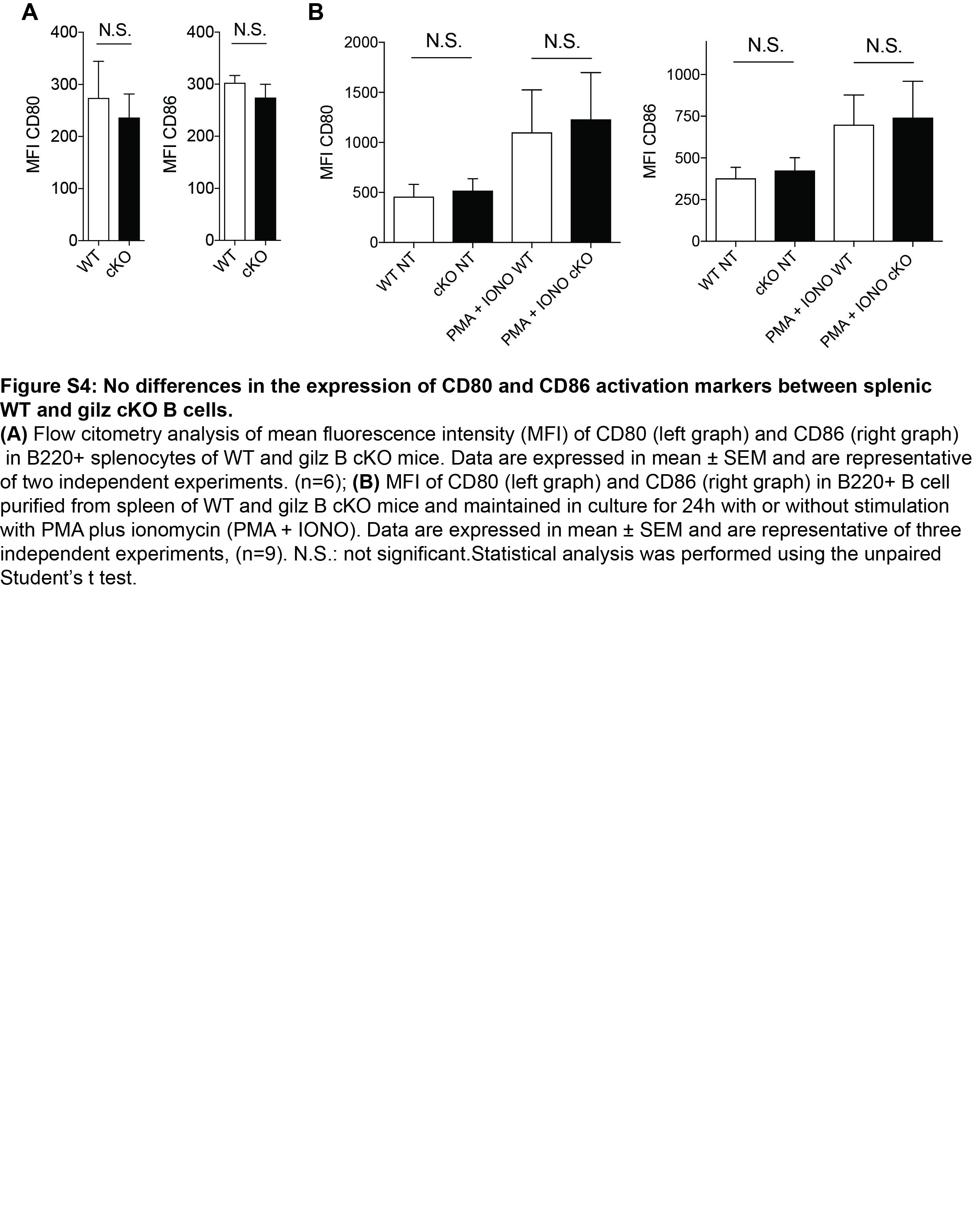

Supplement: Supplementary file 6 [file image_4.jpeg]

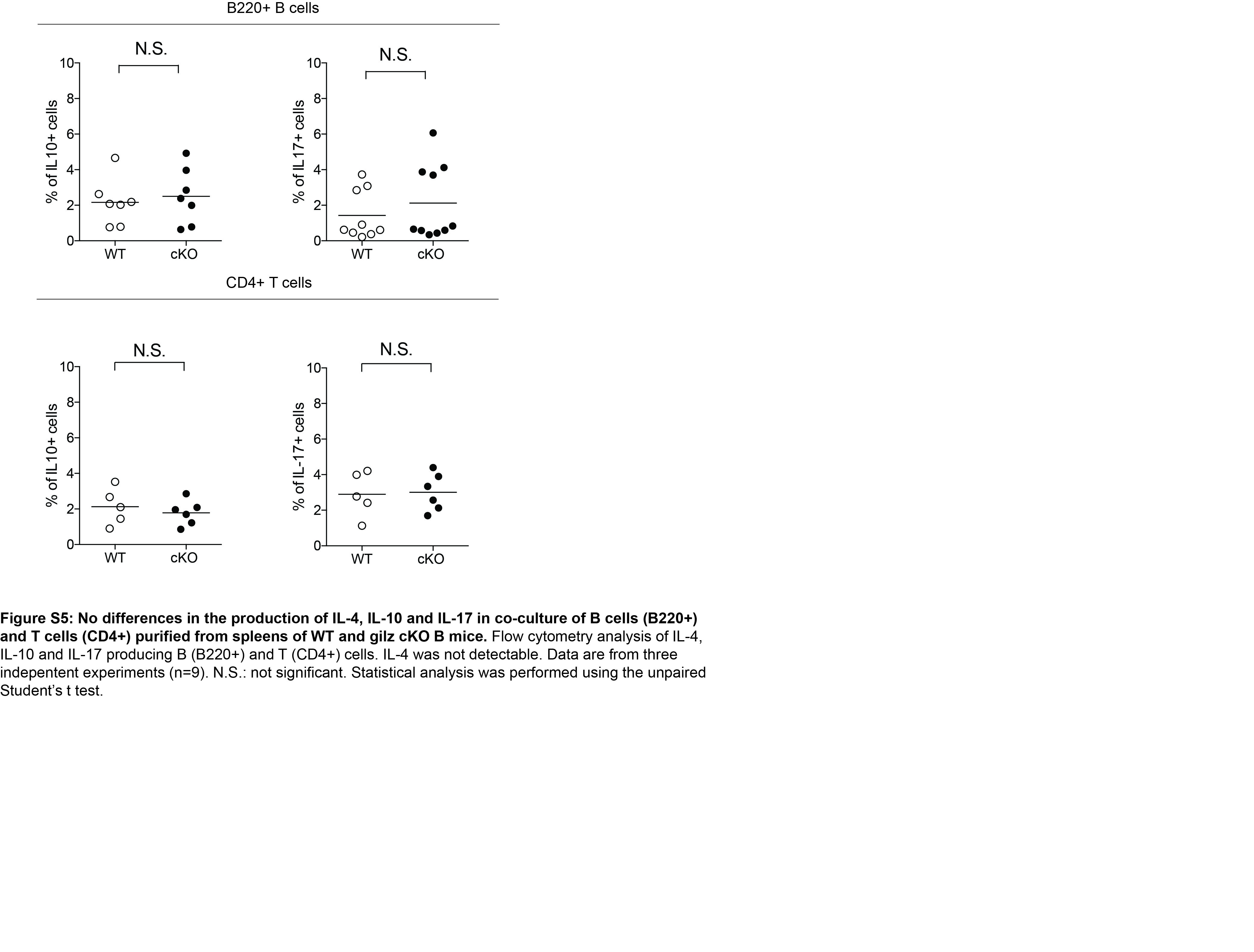

Supplement: Supplementary file 7 [file image_5.jpeg]

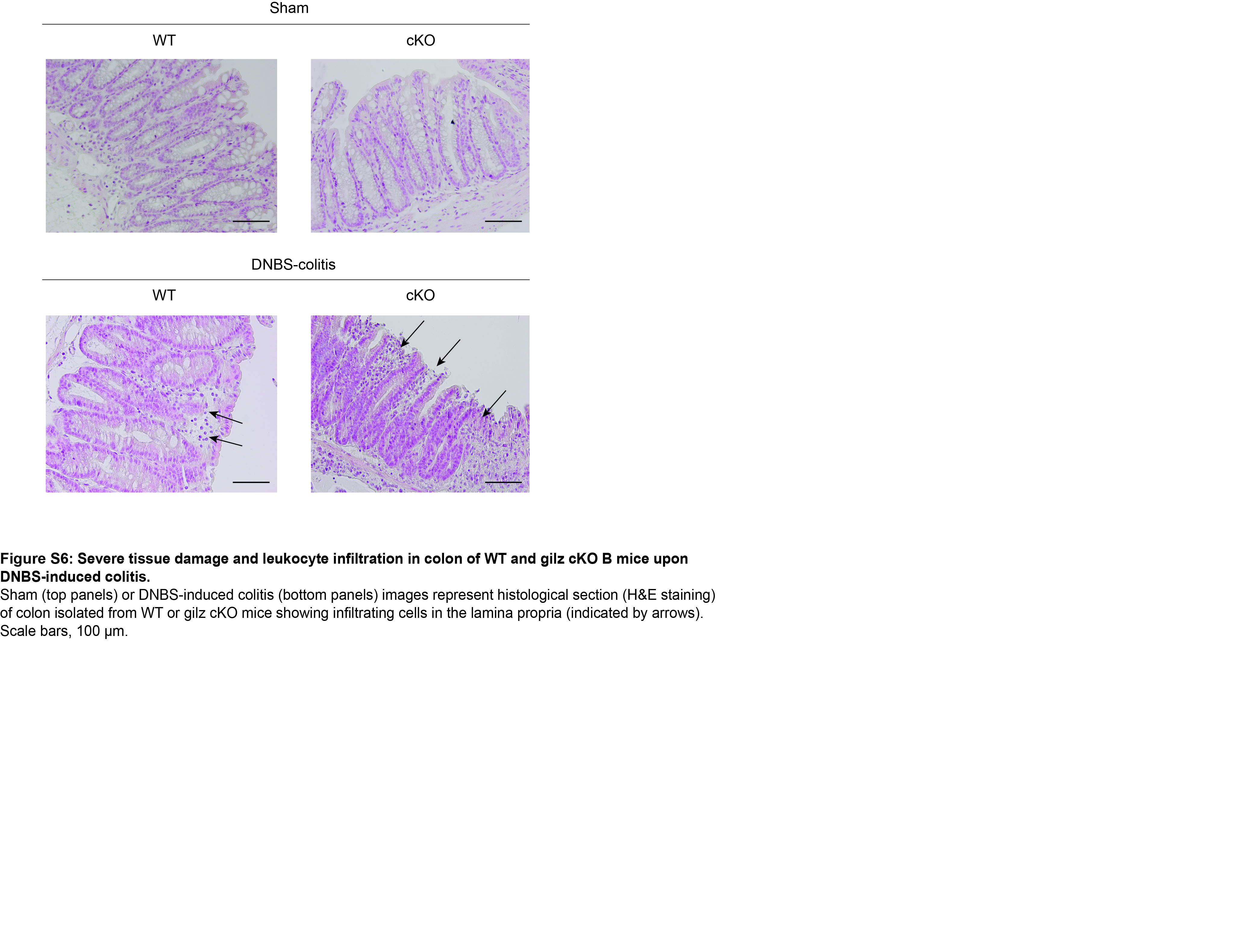

Supplement: Supplementary file 8 [file image_6.jpeg]
